# Neutralizing Oxidized Phosphatidylcholine Reduces Airway Inflammation and Hyperreactivity in a Murine Model of Allergic Asthma

**DOI:** 10.3390/biology13080627

**Published:** 2024-08-17

**Authors:** Jignesh Vaghasiya, Aruni Jha, Sujata Basu, Alaina Bagan, Siwon K. Jengsuksavat, Amir Ravandi, Christopher D. Pascoe, Andrew J. Halayko

**Affiliations:** 1 Department of Physiology and Pathophysiology, University of Manitoba, Winnipeg, MB R3E 3P4, Canada; 2 Biology of Breathing Group, Children’s Research Hospital of Manitoba, Winnipeg, MB R3E 3P4, Canada; 3 Institute of Cardiovascular Sciences, St. Boniface Hospital Albrechtsen Research Centre, Winnipeg, MB R2H 2A6, Canada; 4 Department of Internal Medicine, Max Rady College of Medicine, University of Manitoba, Winnipeg, MB R3E 3P4, Canada

**Keywords:** oxidized phosphatidylcholines, allergic asthma, neutralizing IgM, oxidative stress

## Abstract

**Simple Summary:**

Oxidative stress generates harmful oxygen radicals that activate biomolecules in the lungs of asthmatics. The effects of these mediators are not directly targeted by first-line therapeutics. Using a clinically relevant murine model of allergic asthma, this study reveals that immuno-pharmacological neutralization of oxidized phospholipids in the lung can ameliorate inflammation and asthma symptoms. This suggests that specifically targeting mediators generated by oxidative stress could offer new therapeutic options for asthma to complement conventional therapies.

**Abstract:**

Oxidative stress is associated with asthma pathobiology. We reported that oxidized phosphatidylcholines (OxPCs) are mediators of oxidative stress and accumulate in the lung in response to allergen challenge. The current study begins to unravel mechanisms for OxPC accumulation in the lung, providing the first insights about how OxPCs underpin allergic airway pathophysiology, and pre-clinical testing of selective neutralization of OxPCs in a murine model of allergic asthma. We hypothesized that intranasal delivery of E06, a natural IgM antibody that neutralizes the biological activity of OxPCs, can ameliorate allergen-induced airway inflammation and airway hyperresponsiveness. Adult BALB/c mice were intranasally (i.n.) challenged with house dust mite (HDM) (25 μg/mouse, 2 weeks). Some animals also received E06 monoclonal antibody (mAb) (10 µg) i.n. 1 hr before each HDM challenge. HDM challenge reduced mRNA for anti-oxidant genes (*SOD1*, *SOD2*, *HO-1*, and *NFE2L2*) in the lung by several orders of magnitude (*p* < 0.05). Concomitantly, total immune cell number in bronchoalveolar lavage fluid (BALF) increased significantly (*p* < 0.001). E06 mAb treatment prevented allergen-induced BALF immune cell number by 43% (*p* < 0.01). This included a significant blockade of eosinophils (by 48%, *p* < 0.001), neutrophils (by 80%, *p* < 0.001), macrophages (by 80%, *p* < 0.05), and CD4 (by 30%, *p* < 0.05) and CD8 (by 42%, *p* < 0.01) lymphocytes. E06 effects correlated with a significant reduction in TNF (by 64%, *p* < 0.001) and IL-1β (by 75%, *p* < 0.05) and a trend to diminish accumulation of other cytokines (e.g., IL-4, -10, and -33, and IFN-γ). E06 mAb treatment also inhibited HDM exposure-induced increases in total respiratory resistance and small airway resistance by 24% and 26%, respectively. In conclusion, prophylactic treatment with an OxPC-neutralizing antibody significantly limits allergen-induced airway inflammation and airway hyperresponsiveness, suggesting that OxPCs are important mediators of oxidative stress-associated allergic lung pathophysiology.

## 1. Introduction

Asthma is a chronic lung disease characterized by lung inflammation and airway hyperresponsiveness (AHR) and remodeling [1]. First-line therapy for asthma includes inhaled corticosteroids and β_2_-adrenergic receptor agonists as controller and reliever medications to limit airway inflammation and dilate bronchial airways, respectively [2,3,4,5]. Many asthmatics are refractory to therapy and have uncontrolled symptoms [6,7,8,9], which suggests the presence of underlying persistent airway pathobiology. A feature of treatment-refractory asthma is sustained oxidative stress in the lung that develops in response to an inhaled environment, and is a feature of steroid-refractory asthma pathobiology [10,11,12]. This is associated with the accumulation of inflammation-induced reactive oxygen species (ROS), due in part to impairment of endogenous anti-oxidant systems and effective redox balance [13,14,15,16,17,18]. ROS accumulation is not directly inhibited by inhaled corticosteroids or beta-adrenergic receptor agonists used for standard asthma control. ROS modify protein and lipid molecules, generating new bioactive forms that contribute to the chronicity of inflammation and pathophysiology. Notably, systemic treatment with exogenous anti-oxidant therapeutics has not been effective [19], perhaps due to a need to more specifically target oxidized biomolecules in the lung. Our recent findings show that a panel of oxidized phosphatidylcholines (OxPCs) accumulates in the lungs of human asthmatics after allergen challenge and correlates with clinical indices of airway dysfunction [20,21,22,23].

Oxidative stress modifies phospholipids—including phosphatidylcholine in cell membranes, lipoproteins, and extracellular fluids of the lung—by targeting double bonds in fatty acyl chains to generate numerous fragmented and/or structurally modified forms [24]. OxPCs promote the pathophysiology of atherosclerosis [25], pain-inducing inflammation [26], acute lung injury [27,28], and neurodegenerative diseases [29]. Recent evidence indicates that OxPCs can trigger a number of biological responses that are fundamental to asthma pathophysiology. In human subjects, segmental bronchial challenge with allergens induces the accumulation of panels of OxPCs that associate with AHR and the severity of the late asthmatic response [23,30]. In vitro studies with airway epithelial cells demonstrate that OxPCs promote ROS production, suppress metabolic function of mitochondria, and inhibit wound closure [21]. OxPCs also promote the pro-inflammatory function of human airway smooth muscle cells, inducing the release of IL-6, IL-8, and GM-CSF and the production of oxylipins, including leukotrienes, prostaglandins, and isoprostanes [23]. Moreover, OxPCs directly induce both airway smooth muscle contraction, in part by activating transient receptor potential ankyrin 1 (TRPA1), and bronchospasm in murine lung slice preparations [22,23]. A mechanism for OxPC-induced TRPA1 activation is not yet known; however, a recent study reveals the requirement of cysteine and lysine residues on the N-terminal of channel protein for OxPC agonistic activity [31]. Nonetheless, the specific contributions of OxPCs to asthma pathophysiology in vivo, including airway inflammation and AHR, have not been deciphered in human asthma or in animal models of allergic asthma.

E06 is a murine monoclonal IgM antibody (E06 mAb) that selectively reacts with phosphocholine of oxidized phospholipids, including OxPCs [32,33,34]. It was developed by Dr. J. L. Witztum using apolipoprotein E knockout mice [32]. Studies in vitro and in vivo demonstrate that E06 mAb inhibits pro-inflammatory effects of OxPCs in macrophages [27], reduces infarct size caused by OxPCs in a porcine model of reperfusion injury and protects from OxPC-induced cardiomyocyte death [35], and inhibits OxPC contributions to inflammatory hyperalgesia [26,36]. Additionally, transgenic mice (Ldlr^−/−^-E06-scFv-Tg) that express high plasma levels of the E06 single-chain variable fragment (E06-scFv) are resistant to atherosclerosis [37] and myocardial ischemic-reperfusion injury [35]. Notably, in these animals, E06-scFv is also present in the lung and imparts protection against hyperoxia-induced lung injury [38]. Taken together, these reports indicate that neutralizing the biological activities of OxPCs has potential for therapeutic benefit, but this potential has not yet been evaluated in an animal model of allergic asthma.

To our knowledge, the current study is the first to assess the specific contribution of OxPCs in allergen-induced airway inflammation and AHR in a murine model of asthma. We test the hypothesis that neutralization of OxPCs in the lung using intranasal delivery of E06 mAb is sufficient to prevent allergen-induced airway inflammation and AHR in mice. This approach is unique as it directly tests whether OxPCs may be an important component of persistent lung inflammation that is refractory to mainstay asthma therapies and a previously unknown element of severe asthma pathophysiology.

## 2. Materials and Methods

### 2.1. Chemicals and Antibodies

Methacholine was purchased from Sigma (Oakville, ON, Canada). Pentobarbital sodium injection was from Bimeda-MTC Animal Health Inc. (Cambridge, ON, Canada), and house dust mite (HDM) lyophilized powder was procured from Greer Laboratories Inc. (Lenoir, NC, USA). For our studies, we used HDM Lot #371590, which included 11,375 endotoxin units (EU) per vial. A stock of HDM (2.5 mg/mL) was prepared by dissolving HDM lyophilized powder in saline, and on treatment day each mouse was administered intranasally (i.n.) with 25 µg of HDM diluted into 35 µL of saline. Mouse monoclonal IgM natural antibody E06 mAb was a generous gift from Dr. J. L. Witztum (University of California (San Diego)) [32]. For the flow cytometry immunophenotype assay, we used defined panels of antibodies, as summarized in Table 1.

### 2.2. Animals

Female BALB/c mice (6–8 weeks old) were acquired from the Charles River Laboratories (Senneville, QC, Canada) and housed in individually ventilated cages (Techniplast, Buguggiate, Italy) with access to standard chow food and water ad libitum with 12-h light/dark cycles. All procedures were approved by the University of Manitoba Animal Ethic Committee (Winnipeg, MB, Canada). Following ARRIVE Guidelines, we used a total of 22 animals in this study, and they were randomized, 4 to 5 per treatment group. All animals received an intraperitoneal (i.p.) overdose of pentobarbital sodium (Bimeda-MTC Animal Health Inc., Cambridge, ON, Canada) prior to the procurement of BALF or tissues.

### 2.3. Murine Model of Allergic Asthma and E06 mAb Treatment

As detailed previously, 8–10-week-old BALB/c mice were subjected to repeated intranasal HDM, five days sequential days each week for two weeks [39,40]. For this purpose, individual mice were anesthetized with isoflurane (Forane, Baxter, Deerfield, IL, USA), then administered 25 μg of HDM (in 35 µL of saline) by intranasal instillation (i.n.). The HDM dose (with 3278 130 EU/mg of protein) was selected based on our prior studies, and it is well-tolerated without other adverse health effects on mice [40,41]. Age-matched allergen-naïve animals received intranasal saline (35 µL). For treatment with E06 mAb, animals were administered antibody i.n. (10 µg in 35 µL of saline) 60 min before each HDM challenge, and the control animal received saline alone.

### 2.4. Real-Time PCR for Anti-Oxidant Genes

RNA was extracted from the left lung using the Qiagen RNeasy Kit spin columns. Purity and RNA concentration were measured using Nanodrop 2000 (Thermo Fisher Scientific, Waltham, MA, USA). cDNA was generated from 1µg of total RNA using the BioRad iScript cDNA Synthesis Kit (Hercules, CA, USA). Real-time PCR (RT-PCR) was performed using the BioRad SsoAdvanced Universal SYBR Green Supermix (Hercules, CA, USA) and the BioRad CFX96 Touch Real-Time PCR system (Singapore). Moreover, 18S RNA was used as a housekeeping gene for analysis. Primers for *SOD1*, *SOD2*, *HO-1*, and *NFE2L2* were designed and purchased from IDT Technologies (San Diego, CA, USA) (Table 2). Data were analyzed using ddCT calculation, with the averaged control CT values being used as the reference sample, where a log_2_ value of 0 refers to no change from the reference control.

### 2.5. Lung Function Assessment

Forty-eight hours after the final HDM challenge, each mouse was anesthetized (sodium pentobarbital, 90 mg/kg, i.p.) and underwent tracheostomy with a 20-gauge polyethylene catheter. The tracheal cannula was connected to a flexiVent FX small animal ventilator (SCIREQ Inc., Montreal, QC, Canada), and mice were ventilated (150 breaths/min, tidal volume of 10 mL/kg, and a PEEP of 3 cm H_2_O). Measurement of airway resistance and lung stiffness was performed at baseline and following inhalation of serial challenge with increasing concentrations of nebulized methacholine (Mch) (35 μL, 0–50 mg/mL Mch, 10 sec delivery) [39,42]. To assess lung function, we interrupted low-frequency forced oscillation mechanical ventilation with a volume perturbation signal using the preset flexiVent Prime-8 protocol, enabling measurement of mechanical impedance (Zrs). By fitting Zrs to the constant phase model, flexiVent software (Version 7.6) calculated total respiratory system resistance (Rrs), peripheral tissue damping (G), and tissue elastance or stiffness (H) [43,44]. Each parameter was normalized according to body weight and was calculated as an average of 12 perturbation cycles performed after each Mch challenge.

### 2.6. Bronchoalveolar Lavage Fluid (BALF) Collection

After lung function assessment, BALF was collected using 2 × 1 mL of ice-cold sterile saline. BALF was centrifuged (1100 RPM × 10 min, 4 °C). The supernatant was removed and stored (−80 °C) for subsequent multi-plex cytokine analysis or was used for OxPC extraction and analysis (below). The cell pellet was used for subsequent differential immunophenotype analysis (see below).

### 2.7. OxPC Quantification in BALF

OxPCs were extracted from BALF supernatant using the Folch method, then quantified using ultra-high-performance liquid chromatography–tandem mass spectrometry (LC-MS/MS) (AB Sciex, Framingham, MA, USA) as we have detailed [41].

### 2.8. Flow Cytometry Immunophenotyping of Inflammatory Cells in BALF

BALF cell pellets were re-suspended in 1 mL ice-cold PBS/0.1 mM EDTA, and total leukocyte number was counted (10 μL of cell suspension in a hemocytometer for manual cell number counting). Cell counts were averaged to calculate a total number of cells and normalized per total BALF volume (represented as the number of cells × 10^4^/mL of BALF).

**Figure 1:**
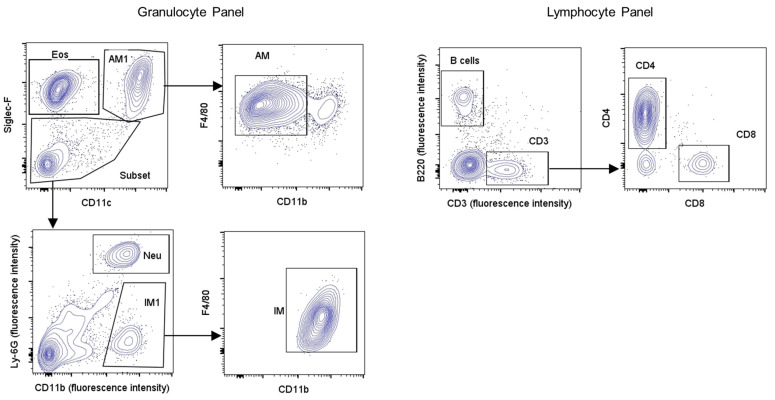
Flow cytometry gating strategy for identification of inflammatory cell populations in BALF. Differential leukocytes in BALF were identified using flow cytometry with the gating strategy depicted above for granulocyte (**left**) and lymphocyte (**right**) identification. Hierarchical gating of non-debris and single cells was followed by differential leukocyte identification based on multiple cell surface markers as mentioned in Table 3. Legend: Eos: eosinophils; Neu: neutrophils; AM: alveolar macrophages; IM: interstitial macrophages.

For cell immunophenotyping, the re-suspended cell pellet was centrifuged (5 min, 1200 rpm, 4 °C), and the resulting pellet was re-suspended in 1 mL FACS buffer (PBS, 0.5% BSA), then for 10 min (on ice) in 100 µL of FC blocker (anti-CD16/CD32, 1 µL in 100 µL of FACS buffer). After washing with 1 mL of FACS buffer, cells were re-suspended in the granulocyte or lymphocyte fluorescent antibody cocktails (30 min, 0.5 µL of each antibody in 20 µL of FACS buffer) (Table 1). After washing cells in FACS buffer, they were slowly re-suspended in 2% paraformaldehyde (400 µL, on ice, 15 min), washed again in FACS buffer, and re-suspended in FACS buffer (500 µL) for storage (4 °C, dark) until analysis using an Attune^®^ NxT acoustic focusing cytometer (Life Technologies Holdings Pte Ltd., Singapore). For multi-color flow cytometry, instrument compensation was established using spleen CD4^+^ cells labeled individually with each antibody (Table 1). Murine spleen cells were isolated from allergen-naïve animals with fine tissue grinding and erythrocyte lysis prior to labeling with individual antibodies as noted above (Table 1). After completing instrument compensation, BALF cells labeled with granulocyte and lymphocyte antibody panels were acquired by cytometer and analyzed using FlowJo (Version 10.8.1) software, with the gating strategies outlined in Figure 1 for cell surface markers (Table 3). The absolute number for each cell type was calculated by converting percentage distribution into absolute number (number of cells × 10^4^/mL of BALF) using respective total cell counts.

### 2.9. BALF Cytokine Analysis

Cytokines and chemokines in BALF supernatant were profiled using the V-PLEX Mouse Cytokine 19-Plex Kit from Meso Scale Discovery (Rockville, MD, USA) following the manufacturer’s instructions. The panel included the following targets: interleukin (IL) -1β, -2, -4, -5, -6, -9, -10, -12p70, -15, -17A/F, -27p28/IL-30, and -33, and IFN-γ, IP-10, KC/GRO, MCP-1, MIP-1α, MIP-2, and TNFα.

### 2.10. Statistical Analysis

Each treatment group consisted of five animals for the positive control HDM challenge protocol, and four animals were included in the E06 treatment group. Depending on the experimental design, data were analyzed as described in the text using a *t*-test, one-way, or two-way ANOVA with Dunnett’s post hoc test using GraphPad Prism version 8.4.3 (San Diego, CA, USA). Data are shown as mean ± SEM unless otherwise specified. *p* < 0.05 was considered statistically significant.

## 3. Results

### 3.1. HDM Challenge Induces Lung Inflammation and Inhibits Expression of Anti-Oxidant Genes

**Figure 2:**
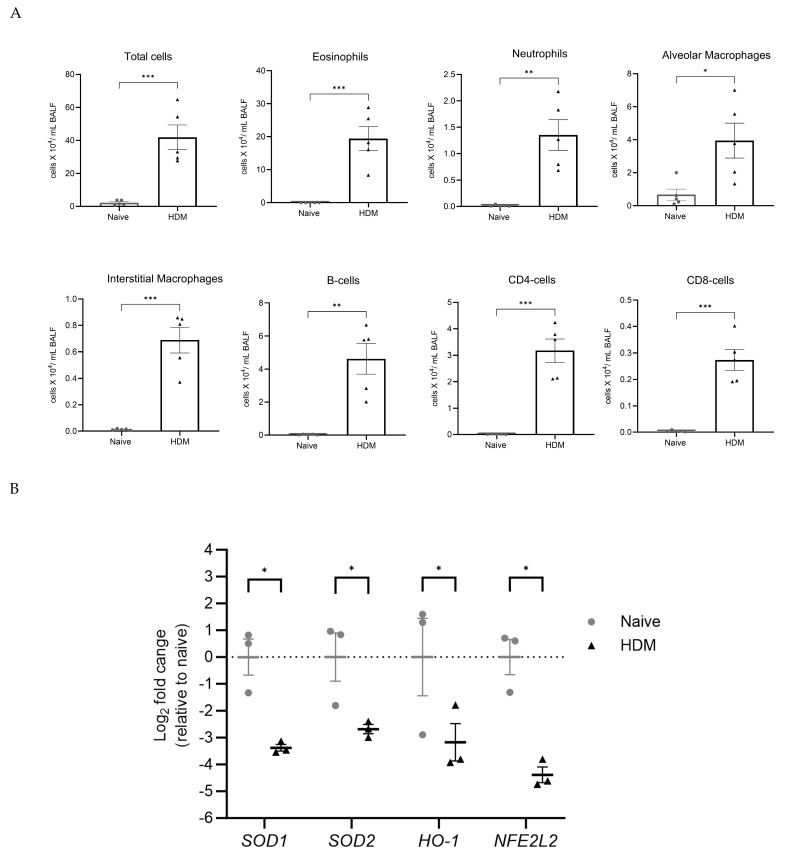
HDM challenge causes inflammatory cell infiltration and redox imbalance in mice lungs. BALF inflammatory cells and lung anti-oxidant genes were assessed at 48 h after the final HDM challenge. (**A**) Total BALF cells were manually counted and normalized to the initial BALF volume. Differential leukocytes were identified using flow cytometry. Scatter dot plots compare the mean ± SEM of total cells and absolute leukocyte counts from five animals per group and were analyzed by a two-tailed *t*-test, * *p* < 0.05, ** *p* < 0.01, and *** *p* < 0.001 HDM vs. naïve. (**B**) Scatter dot plot shows relative Log_2_ fold change in anti-oxidant genes in murine lung tissue from three random animals per group. Data represent mean ± SEM and analyzed by pairwise *t*-test with Holm–Sidak’s multiple comparisons test, * *p* < 0.05 HDM vs. naïve. Legend: *SOD1*: superoxide dismutase 1; *SOD2*: superoxide dismutase 2; *HO-1*: heme oxygenase 1; *NFE2L2*: nuclear factor erythroid 2-related factor 2; HDM: house dust mite.

Repeated HDM challenge increased BALF inflammatory cell number by 17-fold compared to saline-challenged (allergen-naïve) animals (41.8 ± 7.5 × 10^4^ cells/mL vs. 2.2 ± 0.7 × 10^4^ cells/mL, respectively; *p* < 0.001) (Figure 2A). This was associated with a profound increase in eosinophils (*p* < 0.001) and multi-fold and significant accumulation of neutrophils (*p* < 0.01), alveolar macrophages (*p* < 0.05), and interstitial macrophages (*p* < 0.001) (Figure 2A). HDM challenge also caused a significant increase in B-cells (*p* < 0.01) and CD4+ and CD8+ cells (*p* < 0.001) (Figure 2A). Of note, HDM challenge caused a predominant increase in CD4+ over CD8+ cells, with the CD4+/CD8+ cells ratio increasing from 3.0 in the control group to 11.6 in the HDM group. Recent work shows that both CD4+ and CD8+ cells play a central role in HDM-induced allergic asthma in mice [45]. We observed a concomitant decrease (*p* < 0.05) in lung mRNA abundance for the anti-oxidant genes *SOD1*, *SOD2*, *HO-1*, and *NFE2L2* (Figure 2B). This confirms that HDM challenge induces both marked allergic lung inflammation and loss of anti-oxidant gene mRNA that is consistent with redox imbalance that can underpin oxidative stress.

### 3.2. HDM Challenge Induces OxPC Formation in the Lung

**Figure 3:**
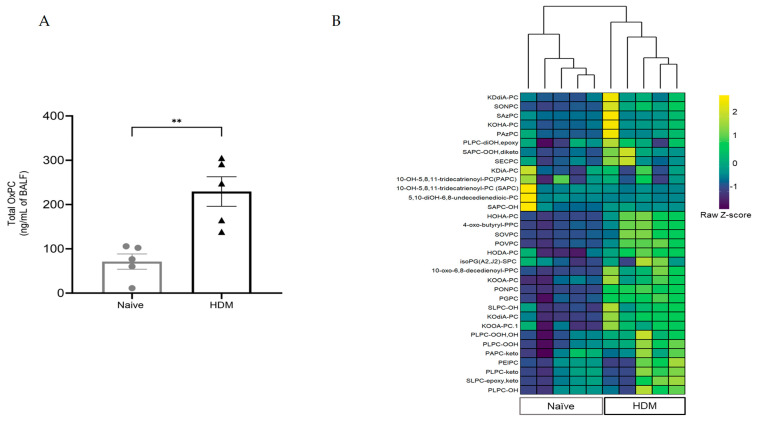
HDM challenge increases OxPC formation in mice’s lungs. BALF OxPCs were measured by LC-MS/MS at 48 h after the final HDM challenge. (**A**) The measured OxPC levels were normalized to the initial BALF volume, and total OxPC is presented as mean ± SEM from five animals per group. The data were analyzed by a two-tailed *t*-test; ** *p* < 0.01 HDM vs. naïve. (**B**) The heatmap compares individual OxPC moieties detected in BALF from naïve and HDM-challenged mice. The heatmap color is based on the row z-score (color key). Agglomerative hierarchical clustering (as shown in the top dendrogram—the vertical length of the dendrogram is proportional to the dissimilarities between the groups or individual animals) shows that the HDM-challenged group is tightly clustered and completely different from the allergen-naïve group based on the individual OxPC moieties level.

LC-MS/MS analysis detected 33 different OxPC moieties in all BALF samples collected from all mice. The total basal concentrations of OxPCs were 71.1 ± 17.2 ng/mL in allergen-naïve animals, but this increased more than 200% in HDM-challenged animals (229.3 ± 33.5 ng/mL of BALF; *p* < 0.01 vs. naïve) (Figure 3A). Agglomerative hierarchical clustering analysis of individual OxPC species in individual BALF samples revealed a distinct increase in the clustered abundance of OxPCs in BALF from allergen-naïve and HDM-challenged mice (Figure 3B).

### 3.3. E06 mAb Treatment Reduces HDM-Induced Infiltration of Lung Inflammatory Cells

**Figure 4:**
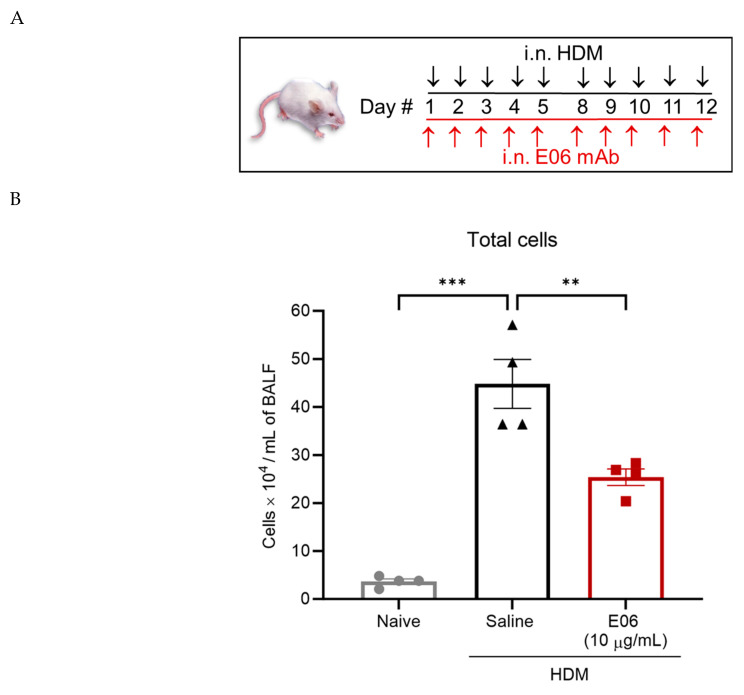

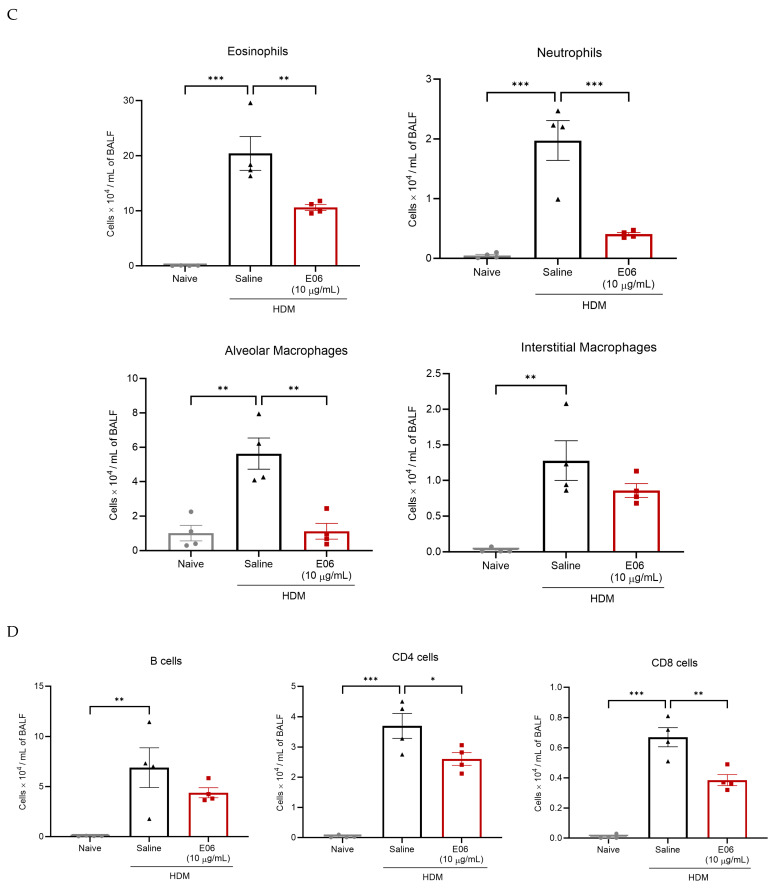

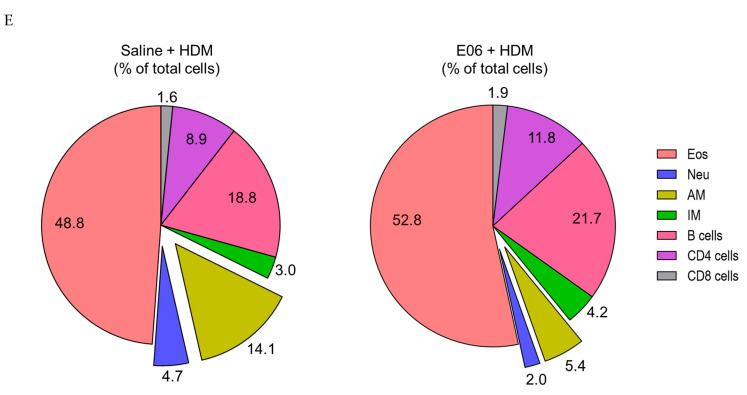
E06 mAb treatment reduces HDM-induced inflammatory cell infiltration in the airways of mice. (**A**) A schematic shows treatment design for E06 mAB and HDM. Black arrows represent HDM (25 μg/day i.n.) challenge and red arrows represent treatment with i.n. E06 mAb (10 μg/day). On each treatment day, E06 treatment occurred 60 min before HDM challenge. BALF inflammatory cells were assessed 48 h after the last treatment day using flow cytometry. (**B**–**D**) Scatter dot plots compare (mean ± SEM) absolute cell counts (total cells and individual leukocytes) between treatment groups (*n* = 4 mice) and are analyzed by one-way ANOVA with Dunnett’s multiple comparisons test, * *p* < 0.05, ** *p* < 0.01, and *** *p* < 0.001 vs. the saline + HDM group. (**E**) Pie charts compare the relative percentage distribution of leukocytes (as a percentage of total cells) between treatment groups. Exploded sections in the chart (Neu and AM) represent the significance difference between treatment groups (** *p* < 0.01 for Neu, * *p* < 0.05 for AM) by paired two-tailed *t*-test. The number in the chart represents the average percentage of four animals per group. Legend: HDM: house dust mite; i.n.: intranasal; Eos: eosinophils; Neu: neutrophils; AM: alveolar macrophages; IM: interstitial macrophages.

E06 mAb treatment significantly reduced HDM-induced total BALF cell counts by 43% (*p* < 0.01, E06-HDM vs. saline-HDM) (Figure 4B). Differential cell analysis showed that the effect of E06 mAb treatment was associated with an ~80% suppression in accumulation of both neutrophils (*p* < 0.001, E06-HDM vs. saline-HDM) and alveolar macrophages (*p* < 0.01, E06-HDM vs. saline-HDM) (Figure 4C), which was reflected in a significant decrease in the percentage of each cell type (*p* < 0.01 for Neu and *p* < 0.05 for AM, comparing E06-HDM vs. saline-HDM) (Figure 4E). E06 treatment was also associated with significantly lower (48%) BALF eosinophil counts (*p* < 0.01, E06-HDM vs. saline-HDM) and a trend for fewer interstitial macrophages (33% lower; *p* = 0.196, E06-HDM vs. saline-HDM) (Figure 4C). Similarly, E06 treatment inhibited HDM-induced infiltration of CD4+ and CD8+ lymphocytes by 30% (*p* < 0.05, E06-HDM vs. saline-HDM) and 42% (*p* < 0.01, E06-HDM vs. saline-HDM), respectively. (Figure 4D). B-cell accumulation appeared to decrease by 36%, but this did not reach statistical significance (*p* = 0.278, E06-HDM vs. saline-HDM) (Figure 4D). The percentage distribution of eosinophils, interstitial macrophages, B cells, and CD4+ and CD8+ cells in BALF was unchanged by E06 treatment (Figure 4E). 

### 3.4. Impact of E06 mAb Treatment on HDM-Induced BALF Cytokines and Chemokines

**Figure 5:**
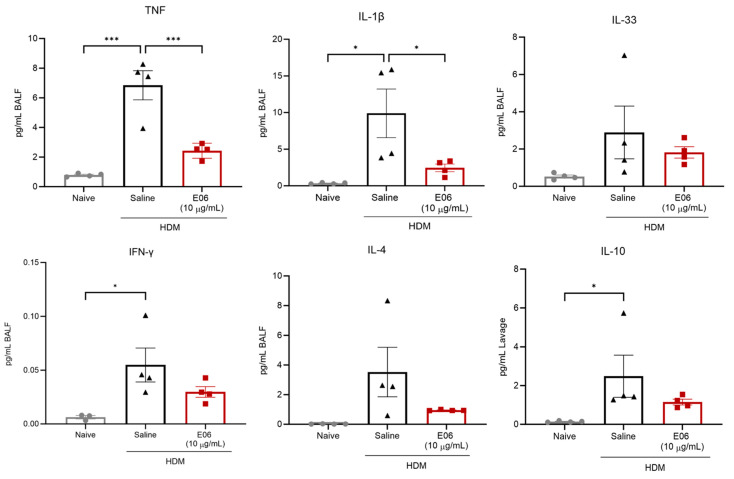
Impact of intranasal E06 mAb treatment on HDM-induced pro-inflammatory cytokine abundance in the airways of mice. BALF cytokines abundance was assessed 48 h after the last treatment. For all data, cytokine level is normalized to initial BALF volume and presented as mean ± SEM from four animals per group. Data were analyzed by one-way ANOVA with Dunnett’s multiple comparisons test, * *p* < 0.05, and *** *p* < 0.001 vs. saline + HDM group.

Mesoscale multi-plex analysis confirmed that allergen challenge led to a significant increase in a number of asthma-associated cytokines and chemokines in BALF (Figure 5 and Supplementary Figure S1). The levels of IL-9, -12p70, -9, and -27p28 were below detection limits across all groups, and though IL-2 and MCP-1 were not consistently detected in BALF from allergen-naïve animals, they were elevated and detected in BALF from HDM-challenged mice (Figure S1).

E06 mAb treatment did result in a 64% inhibition of HDM-induced TNF (2.43 ± 0.25 pg/mL in the E06-HDM group vs. 6.84 ± 0.99 pg/mL in the saline-HDM group; *p* < 0.001) and 75% suppression of IL-1β accumulation (2.45 ± 0.52 pg/mL in the E06-HDM group vs. 9.89 ± 3.33 pg/mL in the saline-HDM group; *p* < 0.05) (Figure 5). E06 mAb treatment was also sufficient for a trend to inhibit the accumulation of HDM-induced IL-33 (37% decrease, *p* = 0.586, E06-HDM vs. saline-HDM), IFN-γ (45% decrease, *p* = 0.201), IL-4 (73% decrease, *p* = 0.191), and IL-10 (54% decrease, *p* = 0.282). E06 mAb treatment was not sufficient to affect the accumulation of IL-2, -5, -6, -17A, or KC/GRO, MIP-1α, MIP-2, MCP-1, and IP-10 in HDM-challenge mice (Supplementary Figure S1).

### 3.5. Impact of E06 mAb Treatment on HDM-Induced Lung Airway Hyperreactivity

**Figure 6:**
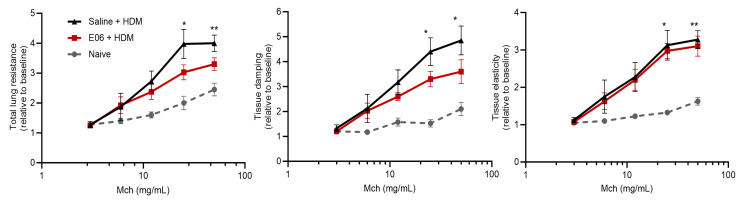
Impact of E06 treatment on HDM-induced airway hyperreactivity in mice. Mouse lung function was assessed 48 h after treatment using a flexiVent small animal ventilator in response to sequential challenge with saline and Mch (3–50 mg/mL). Lung function data [total respiratory resistance (Rrs), tissue damping (G), and tissue elastance (H)] were normalized using respective baseline lung function for each mouse. Data represent mean ± SEM from four animals per group and analyzed by two-way ANOVA with Dunnett’s multiple comparisons test, * *p* < 0.05 and ** *p* < 0.01 vs. naïve controls. Legend: Mch = methacholine.

Consistent with the induction of airway inflammation by repeated challenge with HDM, we also observed an allergen-induced increase in total respiratory resistance (Rrs) (*p* > 0.05 at 25 mg/mL Mch and *p* < 0.01 at 50 mg/mL Mch, comparing saline-HDM vs. naive); small airway resistance (tissue damping (G)) (*p* > 0.05 at 25 mg/mL Mch and *p* < 0.05 at 50 mg/mL Mch, comparing saline-HDM vs. naive); and lung elastance (H) (*p* > 0.05 at 25 mg/mL Mch and *p* < 0.01 at 50 mg/mL Mch, comparing saline-HDM vs. naive) in response to Mch exposure, compared to allergen-naïve animals (Figure 6). In allergen-challenged animals, E06 mAb decreased Rrs and G by 24% and 26%, respectively (Figure 6), revealing a protective trend against allergen-induced airway hyperreactivity (*p* = 0.156 for Rrs and *p* = 0.241 for G at 50 mg/mL Mch, comparing E06-HDM vs. saline-HDM). Interestingly, despite these trends, E06 mAb treatment was seemingly without effect on the increased lung elastance (H) induced by repeated HDM challenge.

## 4. Discussion

This study shows some of the first experimental evidence that the biological activities of OxPCs contribute to airway inflammation and airway hyperreactivity in a murine model of allergic asthma. We used repeated i.n. HDM challenge to induce substantial airway inflammation and hyperreactivity [39], and in so doing, we revealed that this also includes a loss of expression of anti-oxidant genes and the accumulation of the oxidative stress biomarkers, OxPCs, in the lungs. As OxPC accumulation in murine lung parallels our prior observations in human asthmatics [23], we investigated whether OxPCs directly contribute to allergic inflammation and lung pathophysiology. We used a pre-clinical prevention design, treating allergen-challenged mice intranasally with an antibody that neutralizes the biological properties of OxPCs [32]. We report that the monoclonal natural IgM antibody, E06 mAb, is sufficient to limit allergen-induced allergic airway inflammation and hyperreactivity.

Allergen-induced airway inflammation and AHR in humans [46,47] and mice [39,48] is marked by an influx of inflammatory cells, including eosinophils, neutrophils, and CD4+ lymphocytes that are regulated by and contribute to the release of Th1 and Th2 cytokines [48,49]. The murine model of intranasal HDM challenge in our studies exhibits these features. We also demonstrate that allergen challenge mitigates the expression of important anti-oxidant genes—*SOD1*, *SOD2*, *HO-1*, and *NFE2L2*—indicating a disruption of endogenous redox homeostasis in the lung. Impaired anti-oxidant capacity is associated with asthma; for example, SOD activity in lung cells is inversely associated with disease severity, inflammation, and airway hyperreactivity [50,51]. Reactive molecular species such as ROS directly activate structural and immune cells to promote inflammation, but they also modify and bio-activate endogenous molecules, including phospholipids such as phosphatidylcholine, which further accentuate oxidative pathobiology [24,52]. OxPC generation and deposition is an end-result of oxidative stress; thus, they are recognized markers of persistent oxidative biology in neurodegenerative disease [29,52,53] and myocardial injury [54,55]. This is also true for chronic lung disease, as we reported that OxPCs accumulate in BALF from atopic human asthmatics after lung allergen challenge, and they are persistently elevated in BALF individuals who exhibit AHR [23]. Lipidomic profiling of BALF from HDM-challenge mice in the current study is, to our knowledge, the first to reveal that OxPC accumulation in the lung is a predominant feature of pre-clinical murine models of allergic asthma.

OxPCs contribute to inflammation, cytotoxicity, and oxidative stress through multiple mechanisms and thus have the potential to be significant contributors to asthma pathobiology. They can act as damage-associated molecular patterns, activating toll-like receptors on immune cells and endothelial cells [24,56]. They cause endothelial dysfunction, impairing nitric oxide signaling and promoting vascular permeability, properties that have been exploited to generate murine models of acute lung injury [27]. Moreover, OxPCs can act as irritants that activate TRPA1, leading to calcium influx and contraction of airway smooth muscle, airway narrowing, as well as neurogenic pain [22,31]. Oxidative damage and cell membrane disruption by OxPCs promote neuronal cell death pathways [52,57,58]. Inflammation and pro-inflammatory cytokine and chemokine production induced by OxPCs is associated with NF-κB activation and complex kinase networks [23,27,59]. We reported that OxPC activates protein kinase C in human airway smooth muscle cells, and this is associated with cyclooxygenase-2 expression and oxylipin production [23]. Moreover, OxPCs promote ROS generation and inhibit mitochondrial metabolism and wound healing capacity in lung epithelial cells [21]. Lipid uptake, synthesis, and storage in macrophages and adipocytes is modulated by OxPCs, thereby promoting lipid accumulation, foam cell formation in atherosclerotic plaques, and foamy macrophage formation in response to cigarette smoke exposure [60]. Our new observations showing that neutralizing the biological activity of OxPCs in the lungs of allergen-challenged mice is associated with a mitigation of inflammation and lung pathophysiology are consistent with a central role for OxPCs in asthma pathobiology. In addition to OxPCs, other lipid peroxidation end-products such as malondialdehyde (MDA) and 4-hydroxynonenal (4-HNE) are important oxidation-specific epitopes (OSEs) that also contribute to asthma pathobiology and severity [20,61].

Commercially available E06 mAb is used for diagnostic applications to selectively detect E06-reactive OxPCs in various inflammatory diseases [27,53,55,62,63]. E06 selectively binds to the phosphocholine headgroup of oxidized phospholipids, and it does not interact with unoxidized phosphocholine [32,34]. The therapeutic potential of E06 lies in its capacity to neutralize OxPC bioactivity, and there are multiple recent reports of therapeutic properties, including anti-inflammatory, anti-atherogenic, and anti-nociceptive effects, as well as promoting inflammation resolution. Examples include decreased infarct size with intracoronary E06 in a porcine model of ischemia/reperfusion injury [64], increased macrophage uptake of oxidized lipids and lower lung inflammatory markers with intranasal E06 in cigarette smoke-exposed mice [60], and reduced OxPC-induced pain inflammation and hypersensitivity with local E06 application [26,36]. Transgenic mice that express high levels of the single-chain variable fragment of E06 (E06-scFv, a functional E06 component responsible for binding to OxPCs) in plasma are resistant to OxPC-induced inflammatory signaling in atherosclerosis, aortic stenosis, and hepatic steatosis [37]. Notably, in these mice, E06-scFv is expressed in the lung, and the animals are refractory to hyperoxia-induced oxidative stress and lung inflammation [38]. Based on these prior studies, we chose to investigate the effects of lung-delivered E06 mAb (at a dose that is similar to prior reports) over the course of repeated allergen challenges.

A unique observation of our work is that the inflammation-reducing effects of E06 mAb treatment are principally associated with a reduction in neutrophils and alveolar macrophages. This is consistent with known effects of OxPCs on neutrophil and macrophage-mediated inflammation in the lung [27,65,66]. OxPC effects on allergen-induced airway neutrophil and macrophage numbers are also consistent with the reduced levels of several pro-inflammatory cytokines in BALF, including IL-1β (produced by alveolar macrophages and airway epithelium) and Th1 cytokines (TNF and IFN-γ) [67]. We noted a trend for E06 mAb treatment to decrease lung eosinophil, CD4 and CD8 lymphocyte accumulation, and this was paralleled by trends (but not statistically significant) for reduced Th2-defining (IL-4) and epithelium-derived cytokines (IL-33) [67]. IL-33 is an important marker of severe and steroid-refractory asthma [68,69]. Our previous reports [69,70] showed that Th1 cytokines (TNF and IFN-γ) synergistically increase IL-33 expression, and steroids fail to inhibit these effects. Interestingly, in this study, the E06-induced reduction of both TNF and IFN-γ correlates with a trend for IL-33 reduction, suggesting unique effects of OxPCs in asthma pathophysiology. Of note, IL-10, an anti-inflammatory Th2 signal that increases following HDM challenge [39,71,72], also shows a trend to decrease with E06 mAb treatment, an observation that may reflect the declining BALF lymphocyte counts. Overall, E06 mAb treatment shows a general inflammation suppressive effect. An important limitation of our study is that we used a predicted effective dose of E06 mAb based on prior studies with mouse models of other inflammatory diseases. As we did not have sufficient capacity to perform an extensive screen to determine an optimized dose and inhaled delivery modality, our study does not likely demonstrate the full therapeutic potential for lung-delivered E06 mAb.

Our study also assessed the impact of E06 mAb treatment on allergic inflammation-associated lung dysfunction. Repeated HDM challenge results in significantly increased reactivity to Mch, as revealed by measurement of total respiratory resistance and tissue damping, which includes a significant component related to small airway restriction and lung stiffness. E06 mAb treatment resulted in >20% improvement in both total lung resistance and tissue damping compared to HDM-challenged mice. Though not statistically significant, this is a potentially significant clinical effect; however, due to limitations in the availability of the E06 mAb, we were not able to perform a full dose–response study that would have enabled treatment optimization. An interesting observation is that tissue damping may be improved by E06 mAb, suggesting the potential for correcting surfactant dysfunction that may have been induced by HDM challenge [73]. As phosphatidylcholine is a major component of surfactant, this observation suggests that OxPC formation may compromise surfactant function and contribute to lung dysfunction in murine models. To address this possibility, future studies that incorporate advanced surfactometry are warranted, as this will also reveal whether any therapeutic potential of E06 mAb is associated with protective effects on lung surfactant.

Our ability to fully assess the potential magnitude that E06 therapy might hold for allergic airway pathophysiology is limited by the fact that we did not include a comparison group treated with clinically relevant doses of inhaled corticosteroids. A recent study by Lewis et al. [74] reported that 3 mg/kg fluticasone propionate was not sufficient to abrogate inflammation and AHR in mice challenged with mixed allergens. Notably, they observed a 20% reduction in maximum methacholine-induced respiratory resistance, which is equal to or less than the effects of intranasal E06 in our work. They also reported a <50% decrease in CD4+ cells, which mimics the effects of E06 reported here. Interestingly, they also observed that inhaled corticosteroid was without effect on the change in expression of anti-oxidant genes [74]. This suggests that oxidative stress leading to the formation of OxPCs may have been a feature of their allergen-challenged animals, even with corticosteroid treatment. This warrants a future study of co-treatment with corticosteroids and E06, as there appears to be potential for additive or synergistic effects. As E06 does not interact with other OSEs (MDA and 4-HNE), additional effects of anti-oxidants with E06 may reveal an enhancement of therapeutic benefit.

The most significant limitation of the current study relates to the lack of optimization of the dose of E06 mAb we used for repetitive intranasal delivery. We used a single dose (10 µg) in a limited number of animals. E06-scFv TG mice exhibit plasma and lung E06 expression between 20 and 30 µg/mL [37,38]. Testing the full therapeutic potential of exogenously delivered E06 at this concentration range is cost prohibitive. Our observations suggest that future studies are needed using E06-scFv TG mice to understand more specific requirements of OxPCs in a murine model of allergic asthma. E06-scFv TG mice show significantly reduced infiltration of inflammatory cells in aortas in an atherosclerosis study [37]. Thus, studying HDM effects during sustained lung expression of E06 will provide a full understanding of the role of OxPCs in allergen-induced airway inflammation and AHR. In the future, a cost-effective strategy to develop E06 as therapy would be to assess the therapeutic benefit of intranasal E06-scFv synthetic peptide that specifically blocks OxPC bioactivity [37].

## 5. Conclusions

**Figure 7:**
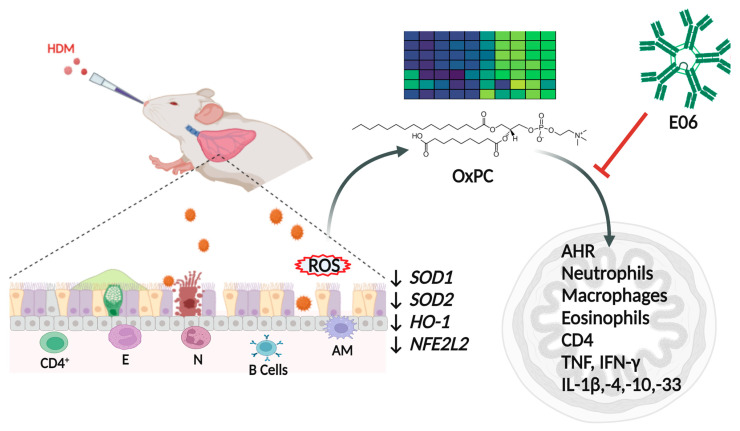
Schematic overview for role of OxPCs in asthma pathobiology. Intranasal HDM challenge in mice causes influx of immune cells (e.g., eosinophils, neutrophils, alveolar macrophages, and CD4^+^ and B-lymphocytes) in the airways. This is associated with a reduction in the expression of important anti-oxidant genes (*SOD1*, *SOD2*, *HO-1*, and *NFE2L2*) in the lungs, leading to a redox imbalance that leads to accumulation of reactive oxygen species (ROS) that generates bioactive oxidative-stress mediators (e.g., OxPCs). E06, a neutralizing antibody, can reduce classical features of airway inflammation and airway hyperresponsiveness. This implicates an important role for OxPCs, oxidative-stress mediators, in allergen-induced asthma pathogenesis. Legend: HDM: house dust mite; *SOD1*: superoxide dismutase 1; *SOD2*: superoxide dismutase 2; *HO-1*: heme oxygenase 1; *NFE2L2*: nuclear factor erythroid 2-related factor 2; E: eosinophils; N: neutrophils; AM: alveolar macrophages; AHR: airway hyperresponsiveness. Created with BioRender.com.

In summary, this is a unique study in its capacity to show that the neutralizing E06 mAb can reduce airway inflammation and hyperreactivity, implicating an important role for OxPCs in allergen-induced lung pathophysiology (Figure 7). However, further research is required to conclusively determine the therapeutic potential of E06 mAb in the context of asthma and other chronic lung diseases. A pathobiological role for OxPC is consistent with our observation that HDM challenge leads to inhibition of anti-oxidation genes, likely generating redox imbalance and oxidative stress. Indeed, OxPC accumulation in the lung is a biomarker for oxidative stress. Overall, this study is a steppingstone for understanding the pathophysiologic role of OxPC as a mediator of oxidative stress in asthma.

## Figures and Tables

**Table 1:** Antibody panels for flow cytometry.

Panel	Antibodies	Concentration (ng/µL)	Source
Compensation	PE anti-mouse CD4	2.0	BioLegend, San Diego, CA, USA
	APC anti-mouse CD4	2.0	
	FITC anti-mouse CD4	5.0	
	APC/Cy7 anti-mouse CD4	2.0	
	PerCP/Cy5.5 anti-mouse CD4	2.0	
	PE/Cy7 anti-mouse CD4	2.0	
Granulocytes	PE/Cy7 anti-mouse/human CD11b	5.0	
	PE anti-mouse CD170 (Siglec-F)	5.0	
	FITC anti-mouse CD11c	12.5	
	PerCP/Cy5.5 anti-mouse F4/80	5.0	
	APC anti-mouse Ly-6G	5.0	
Lymphocytes	PE anti-mouse CD3	5.0	
	FITC anti-mouse CD8a	12.5	
	PerCP/Cy5.5 anti-mouse CD4	5.0	
	APC anti-mouse/human CD45R/B220	5.0	
	PE/Cyanine7 anti-mouse CD335 (NKp46)	5.0	
Fc blocker	Anti-mouse CD16/CD32	5.0	Life Technologies Corp, Carlsbad, CA, USA

**Table 2:** Primer sequence for RT-PCR.

Gene	Forward	Reverse
*SOD1*	ACAATGGTGGTCCATGAGAAA (Sense)	GTTTACTGCGCAATCCCAATC (AntiSense)
*SOD2*	GCAAGGAACAACAGGCCTTA (Sense)	CCCAGTTGATTACATTCCAAATAGC (AntiSense)
*HO-1*	CTAGCCTGGTGCAAGATACTG (Sense)	CAACAGGAAGCTGAGAGTGAG (AntiSense)
*NFE2L2 (NRF2)*	CCATTCCCGAATTACAGTGTCT (Sense)	AGCGAGGAGATCGATGAGTAA (AntiSense)

Legend: *SOD1*—superoxide dismutase 1; *SOD2*—superoxide dismutase 2; *HO-1*—heme oxygenase 1; *NFE2L2*—nuclear factor erythroid 2-related factor 2 or NRF2.

**Table 3:** Cell surface markers for identification of granulocytes or lymphocytes.

Panel	Cell Types	Cell Surface Markers
Granulocytes	Eosinophils (Eos)	CD11c^low^ Siglec-F^+^
	Neutrophils (Neu)	CD11c^low^ Siglec-F^−^ CD11b^high^ Ly-6G^+^
	Alveolar Macrophages (AM)	CD11c^high^ Siglec-F^+^ CD11b^int^ F4/80^+^
	Interstitial Macrophages (IM)	CD11c^low^ Siglec-F^−^ CD11b^high^ F4/80^+^
Lymphocytes	B cells	B220^+^
	CD3 cells	CD3^+^
	CD4 cells	CD3^+^ CD4^+^
	CD8 cells	CD3^+^ CD8^+^

## Data Availability

The original contributions presented in the study are included in the article/Supplementary Material; further inquiries can be directed to the corresponding authors.

## Supplementary Materials

The following supporting information can be downloaded at: https://www.mdpi.com/article/10.3390/biology13080627/s1.

## Abbreviations

AHR (airway hyperresponsiveness), AM (alveolar macrophages), BALF (bronco-alveolar lavage fluid), Eos (eosinophils), E06 mAb (E06 mouse monoclonal natural IgM antibody), FACS (fluorescence activated cell sorting), G (peripheral tissue damping), H (tissue elasticity or stiffness), HDM (house dust mite), *HO-1* (heme oxygenase 1), IM (interstitial macrophages), i.n. (intranasal), Mch (methacholine), Neu (neutrophils), *NFE2L2* (nuclear factor erythroid 2-related factor 2 or NRF2), OxPCs (oxidized phosphatidylcholines), RNS (reactive nitrogen species), ROS (reactive oxygen species), Rrs (respiratory system resistance), *SOD1* (superoxide dismutase 1), *SOD2* (superoxide dismutase 2).
