# Supplementary figures and images for: Neutralizing Oxidized Phosphatidylcholine Reduces Airway Inflammation and Hyperreactivity in a Murine Model of Allergic Asthma
Source: Biology (Basel). 2024 Aug 17;13(8):627. doi: 10.3390/biology13080627 (PMC11351608; doi:10.3390/biology13080627)

## Slide 1
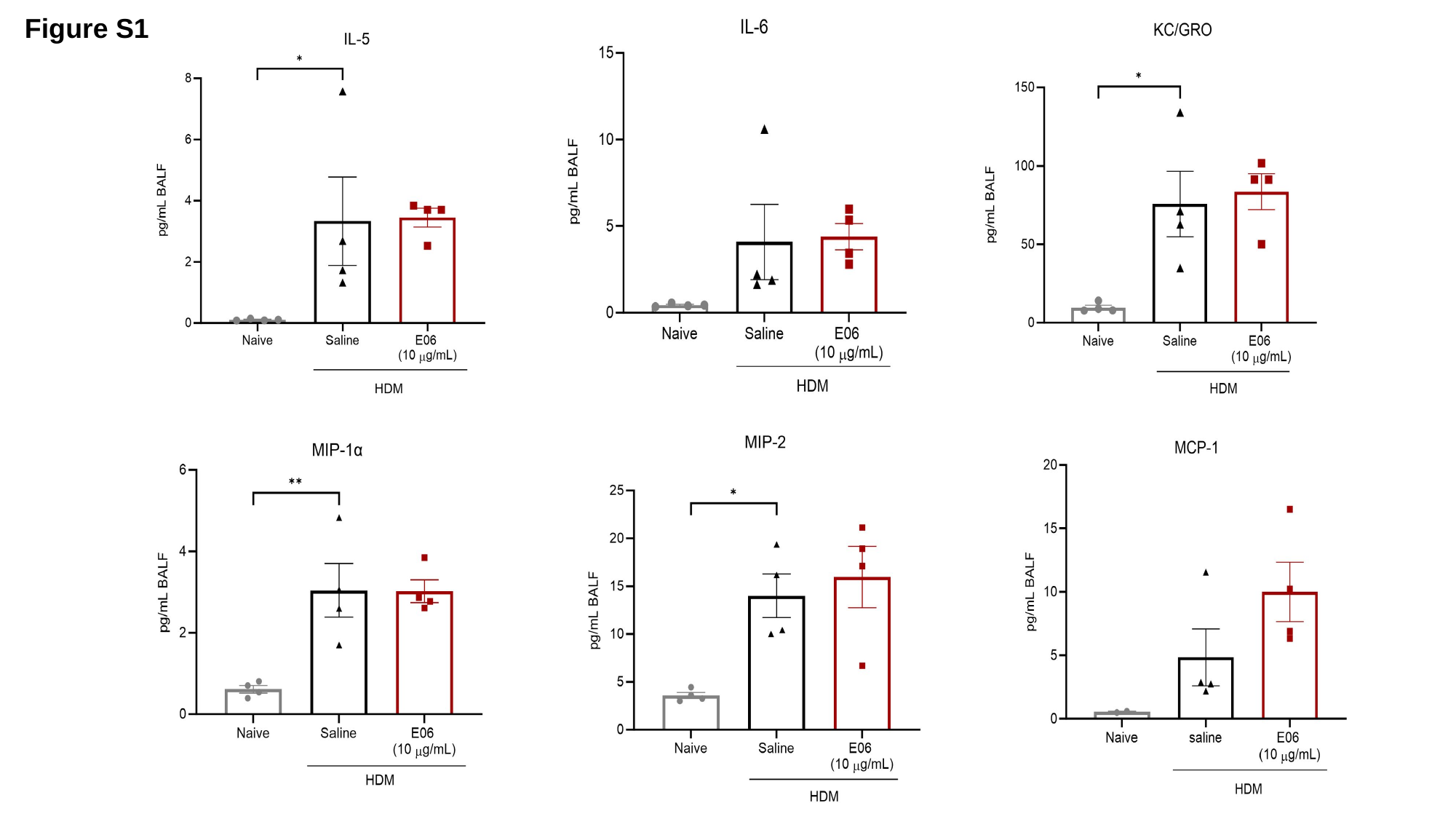

Figure S1

## Slide 2
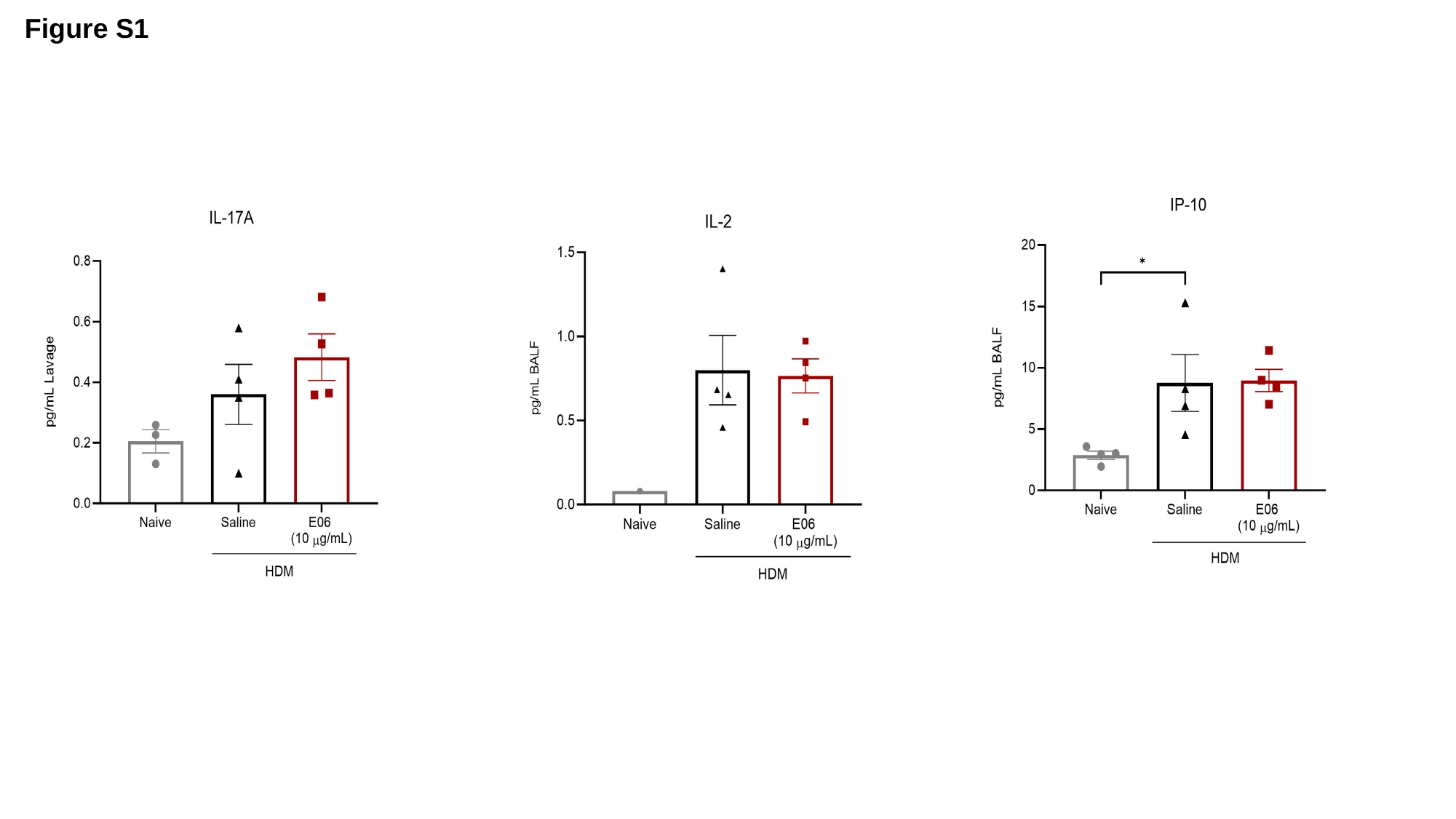

Figure S1

Supplement: Supplementary file 1 [file biology-13-00627-s001.zip › biology-3123584-supplementary.pptx]
